# Safety and aesthetic outcomes of silicone buckles as an implant for anophthalmic sockets: a decade of experience in Japan

**DOI:** 10.1007/s10384-025-01164-9

**Published:** 2025-02-07

**Authors:** Rikako Iwasaki, Yoshiyuki Kitaguchi, Takeshi Morimoto, Hiroshi Shimojo, Takahiro Fujino, Shimpei Komoto, Kohji Nishida

**Affiliations:** 1 Department of Ophthalmology, Osaka University Graduate School of Medicine, 2-2 Yamadaoka, Suita, 565-0871 Osaka Japan; 2 Division of Health Science, Department of Medical Physics and Engineering, Osaka University Graduate School of Medicine, Osaka, Japan; 3 Institute for Open and Transdisciplinary Research Initiatives (OTRI), Osaka University, Suita, Osaka Japan

**Keywords:** Anophthalmic socket reconstruction, Implant, Silicone buckle

## Abstract

**Purpose:**

To investigate the safety and aesthetic outcomes of silicone buckle #506 for anophthalmic socket reconstruction.

**Study Design:**

Retrospective cohort study.

**Methods:**

Patients who underwent enucleation or evisceration at the Osaka University Hospital were retrospectively analyzed. Patients under 18 years old, with < 3 months follow-up, without a prosthesis, or with bilateral surgery were excluded. Aesthetic outcomes were assessed using standardized photographs taken 3 months postoperatively, scored by five independent ophthalmologists for upper eyelid sulcus deepening asymmetry. Scoring was categorized as 0: no noticeable side difference, 1: slight asymmetry, and 2: marked asymmetry characterized by upper eyelid sulcus deepening.

**Results:**

Fifty-nine patients (60 sockets) were analyzed. Thirty-three received silicone buckle implants (1–4 buckles). Implant exposure occurred in two patients (6.1%). Aesthetic scores were assessed in 48 patients. Mean aesthetic scores were 0.97 (no implant), 0.78 (one buckle), 0.68 (two buckles), and 0.42 (three/four buckles) (*p* = 0.123).

**Conclusions:**

Silicone buckle #506 appears to be a safe and feasible option for anophthalmic socket reconstruction. Further studies are needed to optimize aesthetic outcomes and determine the ideal number of buckles.

## Introduction

Anophthalmic socket reconstruction following enucleation or evisceration is crucial for improving the patient’s quality of life. The primary goals of socket reconstruction are to enable the use of a prosthetic eye, maintain facial symmetry, and minimize the psychological impact on the patient.

Commonly used materials for socket reconstruction include porous polyethylene (Medpor), acrylic sphere, silicone sphere, hydroxyapatite, polymethyl methacrylate, and bioceramics [1]. However, in some districts, due to regulatory authorities these materials are not always available for use as anophthalmic socket implants. On the other hand, silicone buckles are a globally authorized material traditionally employed as orbital implants in retinal detachment surgery [2, 3]. We have utilized silicone buckles as anophthalmic socket implants for over a decade in Japan due to regulatory constraints on other materials by the government. There is a previous report on the use of silicone buckles for anophthalmic socket implants. Our report is a further investigation into the use of silicone buckles [4].

The primary aim of this study was to report our experiences regarding the safety of silicone buckles and examine their aesthetic aspects when used in anophthalmic socket reconstruction. Through this investigation, the study will provide a viable source for institutions in which commonly used materials are not readily available due to regulatory restrictions or in situations requiring urgent surgical intervention.

## Methods

### Study Design

**Fig. 1:**
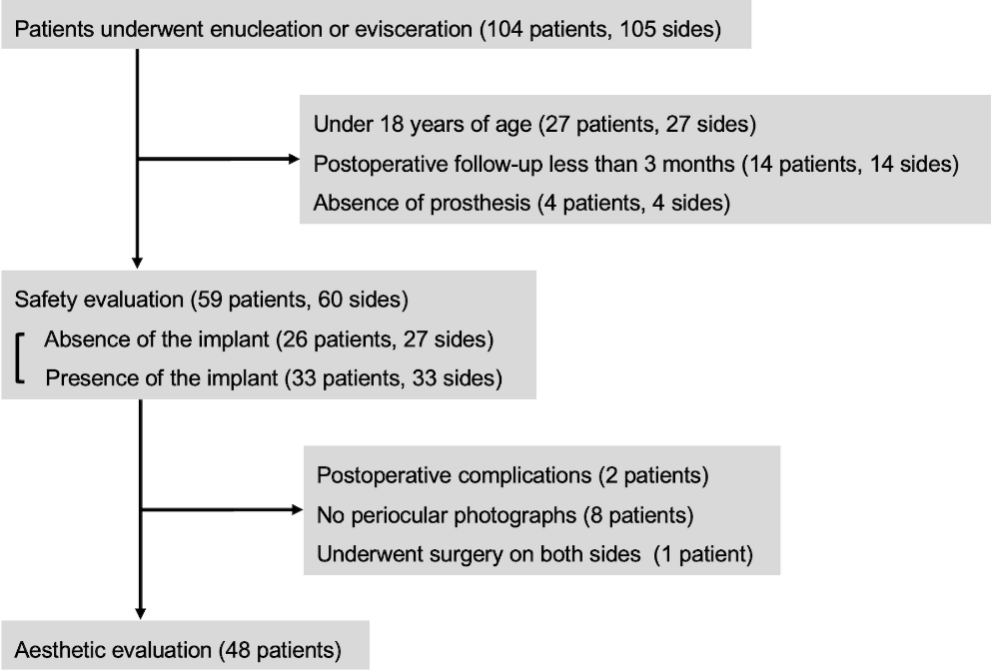
Study flowchart

This was a retrospective chart review of Japanese patients who underwent enucleation or evisceration surgery at the Osaka University Hospital between November 2010 and July 2023. For the safety evaluation, patients under 18 years of age, those with a postoperative follow-up period of less than 3 months, and those who did not use a prosthesis owing to their inability to self-manage were excluded. For the aesthetic outcome evaluation, patients who underwent extrusion of the implant, those without postoperative periocular photographs, and those who underwent bilateral surgery were further excluded (Fig. 1).

This study was approved by the Institutional Review Board of Osaka University Hospital (approval no. 23212) and adhered to the Declaration of Helsinki. The IRB waived the requirement for obtaining informed consent for the research based on the ethical guidelines established by the Japanese Ministry of Education, Culture, Sports, Science, and Technology and the Ministry of Health, Labor, and Welfare. However, an outline of the study was made available for public viewing on the Osaka University Hospital website, giving patients the opportunity to opt out. None of the patients withdrew from the study. While the off-label use of silicone buckles was not pre-approved by the IRB, it was deemed clinically necessary and written informed consent for the procedure was obtained from all patients. Data were anonymized prior to analysis.

### Data Collection

Data regarding age, sex, surgical technique, presence or absence of silicone buckle anophthalmic socket implant, number of silicone buckles #506 used, postoperative complications, and periocular photographs taken 3 months postoperatively were extracted from the medical records.

### Surgical technique

**Fig. 2:**
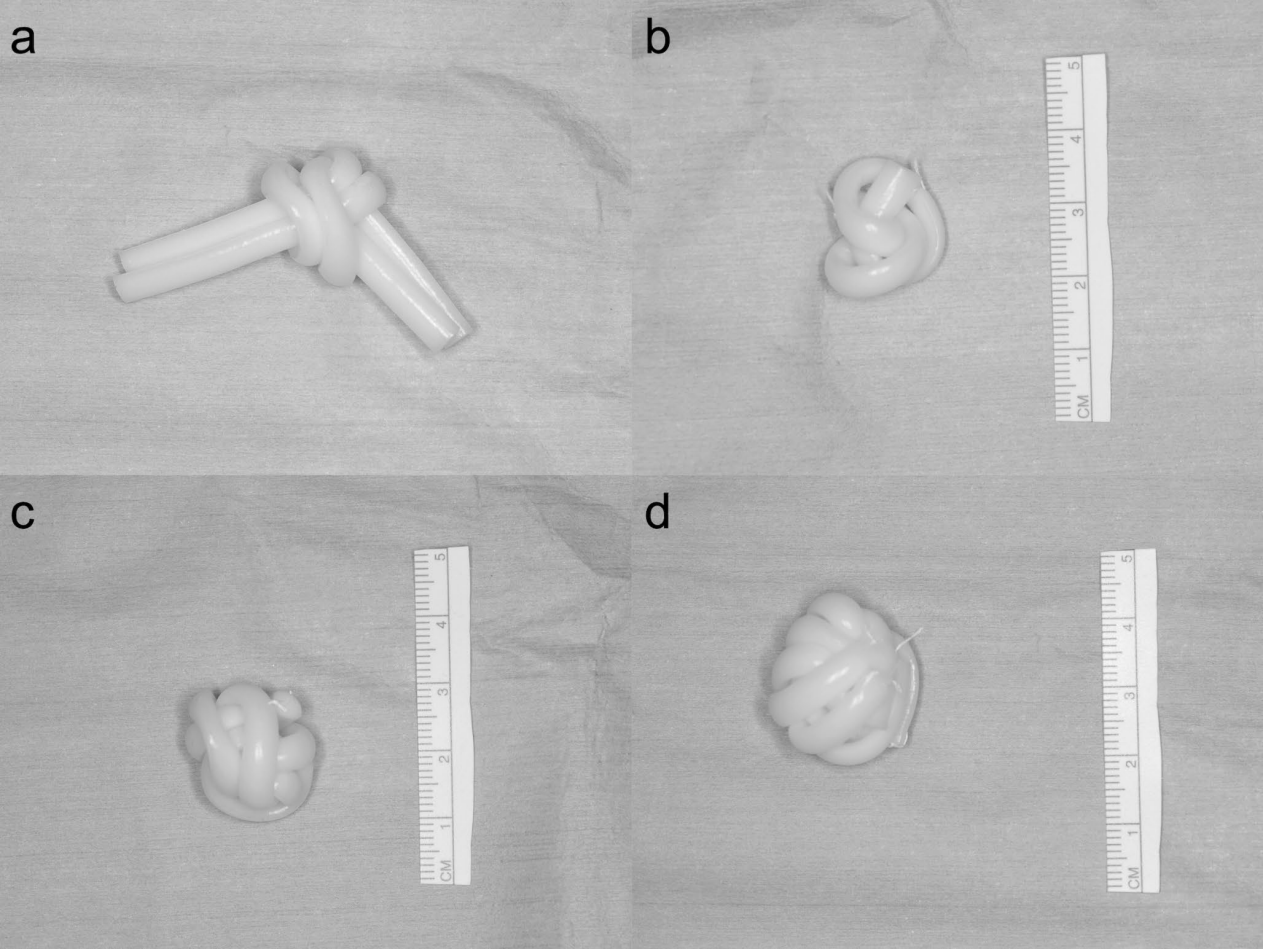
Technique for shaping silicone buckles into a spherical form for use as an anophthalmic socket implant. a Silicone buckles tied into a ball knot (two buckles). b Sphere-shaped implant with one silicone buckle. c Sphere-shaped implant with two silicone buckles. d Sphere-shaped implant with three silicone buckles

All surgery was performed by two surgeons (T.M. and Y.K.). Initially, the implants were prepared by tying a silicone buckle into a ball knot. When using multiple buckles, they were all bundled together and secured with a ball knot. The ends of the silicone buckle knots were fastened to the knot area using 5 − 0 Dacron, tied into a spherical shape (Fig. 2). The method of preparation of the implant was standardized and shared between two surgeons to minimize variation in surgical outcomes.

All enucleations were performed under general anesthesia. A 360° conjunctival peritomy was performed using Wescott scissors. Tenon’s fascia was opened in all four quadrants. The rectus muscles were secured with locking sutures and transected at their insertions. After the oblique muscles were transected, the optic nerve and perineural tissues were excised, and the globe was enucleated. Spherical buckles were inserted into the intraconal space, and the extraocular muscles were attached to each other by suturing directly to the implant using 6 − 0 vicryl sutures. Finally, Tenon’s capsules and the conjunctiva were closed layer-by-layer.

Eviscerations were performed under local or general anesthesia. Retrobulbar anesthesia with 2% lidocaine was used for local anesthesia. A 360° conjunctival peritomy was performed using Wescott scissors, and the sub-Tenon’s fascia was dissected bluntly using Stevens scissors to expose the retrobulbar area. The cornea was removed using Westcott scissors by circumferentially incising the sclera, posterior to the surgical limbus. The uvea was then separated from the sclera, and ocular contents were aspirated using a 3 mm Frazier suction cannula. In cases where a single buckle sphere was inserted, the implant was inserted without making an additional incision in the sclera. When more than two silicone buckles were inserted, the following method was used. A circumferential incision was made in the sclera around the optic disc using a No. 11 scalpel blade and the optic nerve was cut. Two sclerotomies were performed inferonasally and superotemporally from the limbus to the posterior equator of the eyeball, using Stevens scissors. Two sclerotomies were performed, inferotemporally and superonasally, at the posterior pole. By making radial incisions in the sclera as described above, it is easier to provide space for the insertion of the anophthalmic socket [5]. After the insertion of the spherical buckles, two parts of the sclera were overlapped anterior to the socket implant. Finally, the surfaces were aligned and sutured in front of the socket implant using 5 − 0 PDS. Tenon’s capsule and conjunctiva were closed layer-by-layer.

### Aesthetic outcomes measures

**Fig. 3:**
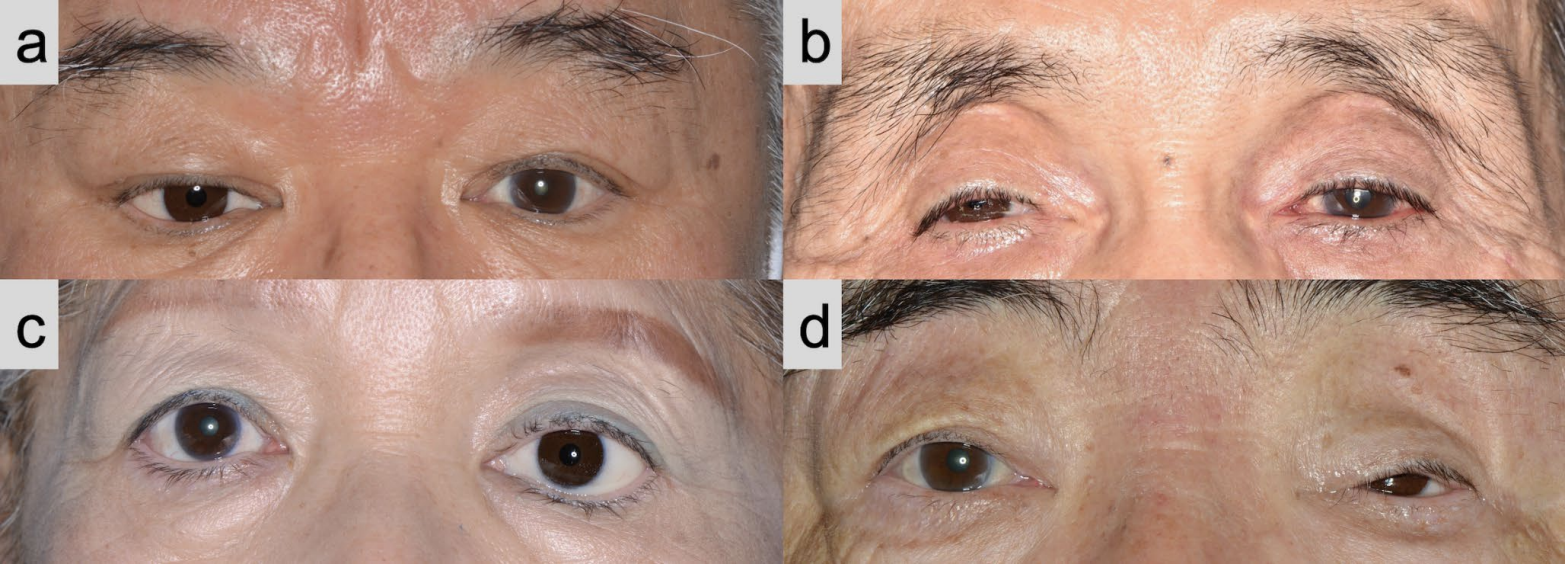
Typical periocular photographs in each aesthetic category Regarding deepening of the upper eyelid sulcus: a, b no noticeable side difference, 0; c not severe but noticeable asymmetry, 1; and d marked asymmetry, 2

Aesthetic scoring was independently performed by five ophthalmologists by reviewing 3-month postoperative periocular photographs regarding the asymmetry of superior sulcus deepening between the operated and non-operated sides. The aesthetic score was categorized as 0 for no noticeable side differences, 1 for not severe but noticeable asymmetry, and 2 for marked asymmetry (Table 1; Fig. 3). The average score of the five ophthalmologists was considered as the final score. To compare the distribution of final scores based on the number of silicone buckles used, the final scores were compiled and analyzed for each buckle count.

**Table 1:** Assessment of aesthetic results based on the asymmetry between the operated and non-operated sides

Score	Findings
0	No noticeable side difference.
1	A slight asymmetry.
2	A marked asymmetry characterized by a deepening of the upper eyelid sulcus.

### Statistical analyses

Data are presented as mean values with accompanying standard deviation. The Kruskal-Wallis test was employed to analyze the relationship between the aesthetic outcome scores and number of silicone buckles used. A p-value of less than 0.05 was considered statistically significant.

## Results

We initially identified 104 patients (105 sides) who underwent enucleation or evisceration. For the safety evaluation, 59 patients (60 sides) were included. The average patient age was 67.2 ± 15.9 years, including 36 (61.0%) men and 23 (39.0%) women. The average postoperative follow-up was 30.0 ± 28.9 months (Table 2).

**Table 2:** Demographic characteristics of the patients

Characteristics	
Number of patients (sides)	59 (60)
Age (years, mean ± SD)	67.2 ± 15.9
Sex (male/female, %)	36/23
Postoperative follow-up (month, mean ± SD)	30.0 ± 28.9
Anophthalmic socket implant (presence/absence, %)	33/26

SD; standard deviation

Among these patients, 18 underwent enucleation and 41 underwent evisceration. For enucleation, the causative diseases included choroidal malignant melanoma (15 sides) and panophthalmitis (three sides). The causes of evisceration were painful phthisis bulbi (14 sides), ruptured eyeball (12 sides), corneal perforation due to infection (10 sides), and infectious endophthalmitis (five sides).

Thirty-three (55.9%) patients underwent silicone buckle anophthalmic socket implantation, whereas 26 (44.1%) did not. The number of silicone buckles #506 used during surgery was: one in 12 (20.3%), two in six (10.2%), three in 12 (20.3%), and four in three (5.1%) patients (Table 3). The number of silicone buckles to be used was determined based on several factors. Insertion was avoided in cases of panophthalmitis or fungal endophthalmitis owing to the heightened risk of infection. Additionally, the decision against using silicone buckles was made when, based on the patient’s age and preferences the disadvantages due to the risk of exposure surpassed the aesthetic benefits. The initial protocol specified the insertion of a single silicone buckle. However, it became apparent that many patients still showed hollowing in the upper eyelid with a single silicone buckle postoperatively. To address this issue, the surgical technique was gradually modified to include the insertion of two or three silicone buckles. Moreover, in a few patients with high myopia and longer axial length, four silicone buckles were inserted to achieve optimal results.

**Table 3:** Number of silicone buckles #506 inserted

Number of silicone buckles used	Patient *n* (%)	Sides *n* (%)
0	26 (44.1)	27 (45.0)
1	12 (20.3)	12 (20.0)
2	6 (10.2)	6 (10.0)
3	12 (20.3)	12 (20.0)
4	3 (5.1)	3 (5.0)

**Fig. 4:**
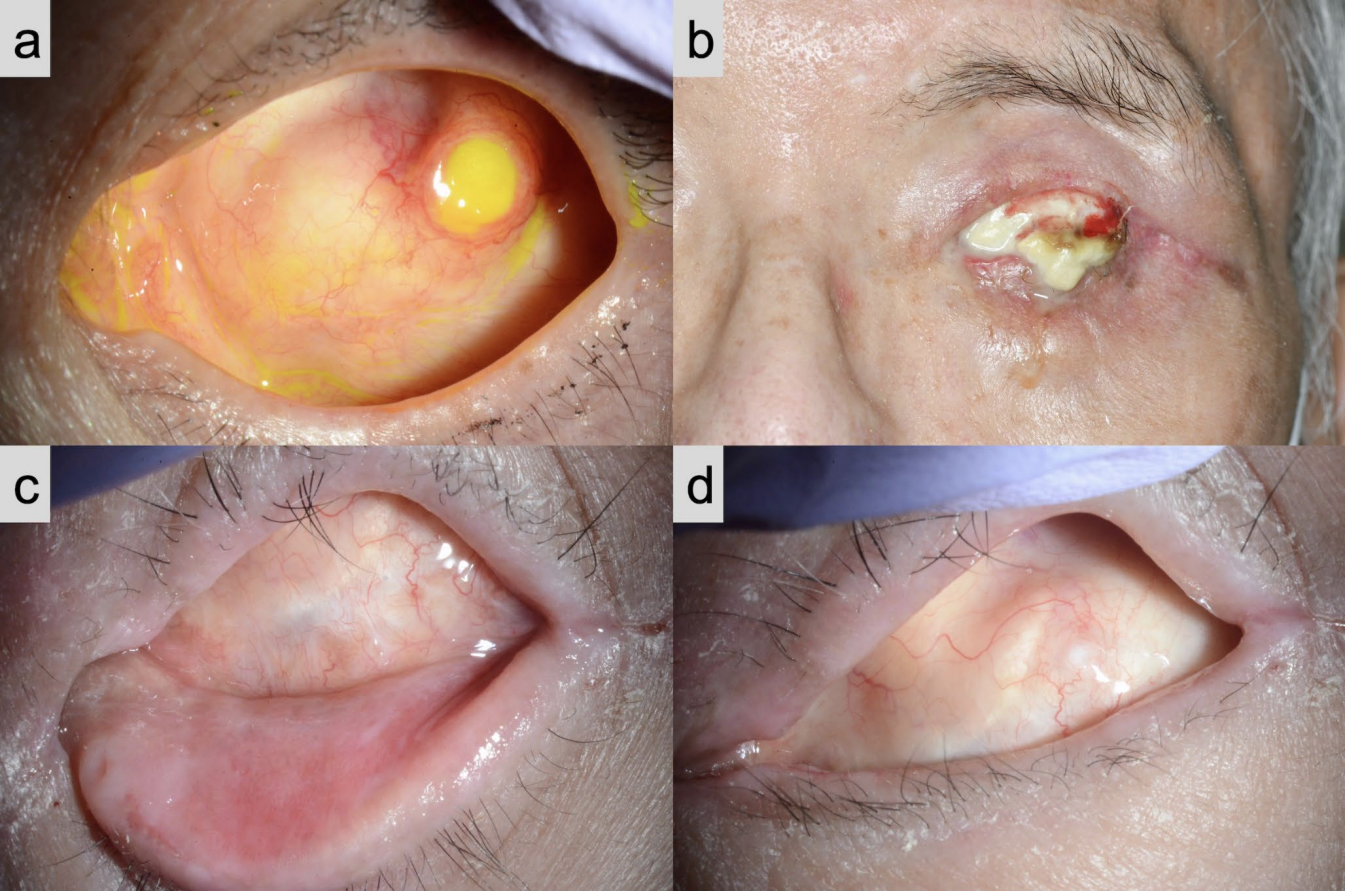
Photographs of conjunctival sacs in different patients a Conjunctival sac with extruded implant in a patient who underwent enucleation. b Conjunctival sac with an infected implant in a patient who had severe lagophthalmos owing to facial nerve paralysis. c, d Conjunctival sac in a patient with no complications

Exposure of the implant occurred in two (6.1%) patients. In one patient who underwent enucleation, the extruded implant was initially managed by debulking and conjunctival suturing (Fig. 4a). One patient who presented with severe facial nerve paralysis underwent evisceration and four silicone buckles were inserted. However, the implant required removal because of implant infection (Fig. 4b). In the other patients, the conjunctival sac was not deformed, although the implant was not smooth in shape (Fig. 4c, d).

**Fig. 5:**
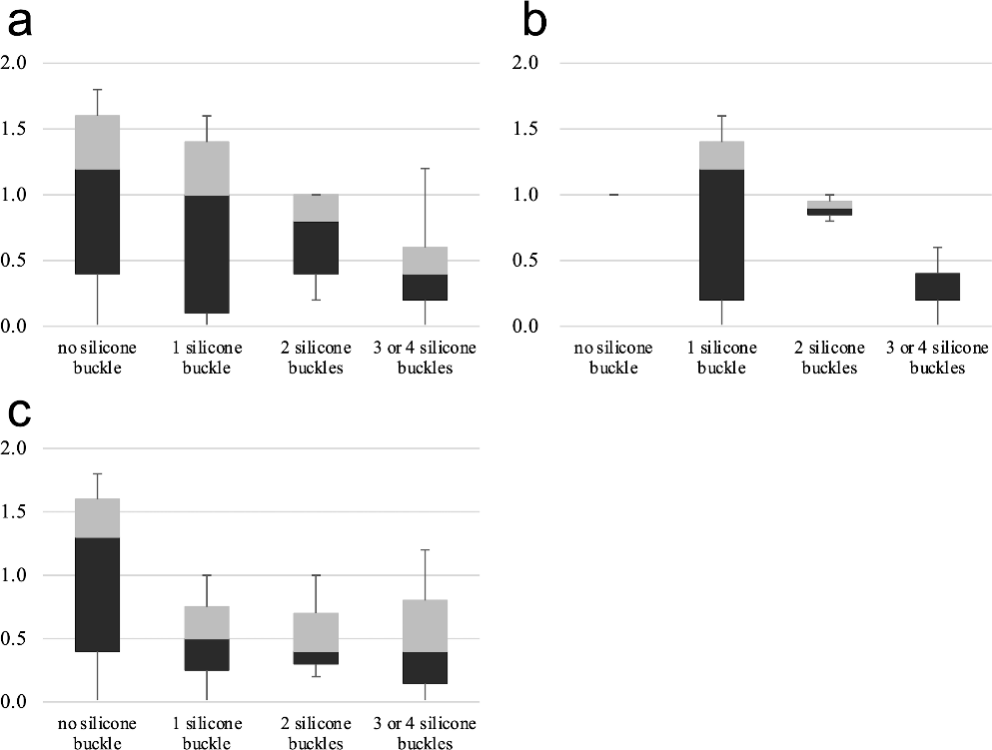
Distribution of the aesthetic results based on the number of silicone buckles a Aesthetic scores of forty-eight patients (including both enucleation and eviceration). b Aesthetic scores of patients who underwent enucleation. c Aesthetic scores of patients who underwent evicerationor

After excluding 11 patients from the cohort for safety evaluation, we assessed the postoperative aesthetic scores of the remaining 48 patients (Fig. 1). Among these patients, 17 underwent enucleation and 31 underwent evisceration, and the aesthetic evaluation was conducted separately according to the surgical procedure. The distribution of aesthetic scores for each number of silicone buckles is shown in Fig. 5. The mean aesthetic scores were: 0.97 without an implant, 0.78 with one silicone buckle, 0.68 with two buckles, and 0.42 when three or four silicone buckles were used. Despite these numerical differences, the variations in scores across the groups were not statistically significant (*p* = 0.123). In the enucleation group, the mean aesthetic score was 1.0 without implant, 0.84 with one silicone buckle, 0.90 with two, and 0.32 when three or four silicone buckles were used (*p* = 0.422), while in the evisceration group, 0.97 without implant, 0.50 with one silicone buckle, 0.53 with two, and 0.49 when three or four silicone buckles were used (*p* = 0.270).

## Discussion

Silicone buckle #506 was successfully used as a material for anophthalmic socket implant. The optimal implant material for the anophthalmic socket should resist infection, provoke minimal or no inflammation, and result in minimal rates of exposure. Silicone buckles have been widely approved for their reliability and suitability in retinal detachment surgery. The long-term stability of this material contributes to its successful use as an anophthalmic socket implant as well as scleral implant for retinal procedures.

Porous polyethylene, acrylic sphere, silicone sphere, hydroxyapatite, polymethyl methacrylate, and bioceramics are widely used materials for anophthalmic implants, for which the extrusion rate is reported. Reports indicate the extrusion rates ranging from 3.7 to 8.3% [6–9], 0.60–1.0% [10, 11], 0.84–18% [12–14], 1.2–6.7% [7, 15–18], 7.1% [19], and 7.0–9.1% [20–22]. In this study, the extrusion rate of the silicone buckle sphere was 6.1%, similar to the rates observed for other materials.

One patient whose ophthalmic implant was extruded was post-enucleation. The rate of exposure is higher after enucleation than after evisceration because the anophthalmic implant is not encased by the sclera [23]. To address this problem, wrapping anophthalmic socket implants with vicryl mesh is reported to lower the exposure risk [24]. Another patient had severe lagophthalmos secondary to facial nerve paralysis, potentially associated with a condition prone to infection that led to corneal perforation and evisceration. This patient also suffered from neurotrophic keratopathy due to trigeminal nerve paralysis. The condition was prone to worsening dry eyes, and the poor ocular surface environment was thought to have caused exposure of the implant and subsequent infection.

The use of multiple silicone buckles increases the volume and diameter of the sphere-formed implants. Considering that the silicone buckle #506 has an oval cross-section with a major axis 5 mm, a minor axis 3 mm and a length of 100 mm, its volume is calculated as 1178 mm³. When this volume is reconfigured into spherical forms, the estimated diameters are 13.1 mm for a single buckle, 16.5 mm for two, 18.9 mm for three, and 20.8 mm for four buckles. However, the actual measured diameter was larger than the predicted value: 18 mm for a single, 20 mm for two, 22 mm for three, and 24 mm for four buckles, likely owing to a gap in forming a spherical shape. Determining the implant diameter by choosing a size 2 mm smaller than the axial length in normal to myopic eyes and reducing it by an additional 1 mm in cases of evisceration or hyperopia, reduces the risk of clinically and aesthetically unacceptable superior sulcus deformity and enophthalmos in 85% of patients [25]. Therefore, it is possible that the number of inserted silicone buckles can be determined according to the axial length.

Our analysis revealed a trend of improving mean aesthetic scores with an increasing number of silicone buckles (0.97 without implant, 0.78 with one buckle, 0.68 with two, and 0.42 with three/four buckles). However, these differences did not reach statistical significance (*p* = 0.123). This lack of statistical significance may be due to several unmeasured patient characteristics. For example, pre-existing orbital anatomy, such as naturally deep-set eyes, could mask the effect of the implant by reducing the perceived asymmetry, even without an implant. Other factors, such as eyelid position and periorbital soft tissue volume, might also influence the aesthetic outcome. To address these limitations in future studies, more objective assessment methods, such as image analysis techniques to quantify sunken areas (e.g., pixel brightness measurements) [26], could be employed. Furthermore, incorporating patients’ subjective assessments of aesthetic outcomes alongside objective evaluations would provide a more comprehensive understanding of the postoperative results [27–29].

This study had several limitations. First, this was a retrospective study with a limited sample size. Larger prospective studies are warranted in the future. Second, the follow-up period was relatively short, with 3 months as the shortest period. A longer follow-up is required to confirm the risk of late complications. Third, there were missing data owing to the retrospective nature of the study, which may have affected the accuracy of the exposure rate. While patients lost to follow-up likely represent uncomplicated cases, the possibility of unreported complications treated elsewhere cannot be excluded.

In summary, our findings suggest that the use of silicone buckles as socket implants is safe and may be a valid option. However, future studies are needed to determine complications and long-term outcomes. Further research with larger sample sizes is needed to determine whether adjusting the number of silicone buckles indeed leads to optimal aesthetic results.
